# A Computational Strategy for Protein Function Assignment Which Addresses the Multidomain Problem

**DOI:** 10.1002/cfg.208

**Published:** 2002-10

**Authors:** A. J. Pérez, A. Rodríguez, O. Trelles, G. Thode

**Affiliations:** 1 Genetics Department University of Málaga Málaga 29071 Spain; 2 Computer Architecture Department University of Málaga Málaga 29071 Spain

## Abstract

A method for assigning functions to unknown sequences based on finding correlations between short signals and functional annotations in a protein database is presented. This approach is based on keyword (KW) and feature (FT) information stored in the SWISS-PROT database. The former refers to particular protein characteristics and the latter locates these characteristics at a specific sequence position. In this way, a certain keyword is only assigned to a sequence if sequence similarity is found in the position described by the FT field. Exhaustive tests performed over sequences with homologues (cluster set) and without homologues (singleton set) in the database show that assigning functions is much ’cleaner’ when information about domains (FT field) is used, than when only the keywords are used.


**Comparative and Functional Genomics**
*Comp Funct Genom* 2002; **3**: 423–440.
Published online in Wiley InterScience (www.interscience.wiley.com). **DOI:** 10.1002/cfg.208

**Research Article**

### A computational strategy for protein function assignment which addresses the multidomain problem

A. J. Pérez[1]*, A. Rodríguez[2], O. Trelles[2] and G. Thode[1]
[1] *Genetics Department, University of Málaga, 29071 Málaga, Spain*
[2] *Computer Architecture Department, University of Málaga, 29071 Málaga, Spain*

**Correspondence to:*
*A. J. Pérez, Genetics Department, University of Málaga, 29071 Málaga, Spain.*
*E-mail: antoniojperez@uma.es*

Received: 15 March 2002
Accepted: 12 August 2002

### Abstract

**A method for assigning functions to unknown sequences based on finding correlations between short signals and functional annotations in a protein database is presented. This approach is based on keyword (KW) and feature (FT) information stored in the SWISS-PROT database. The former refers to particular protein characteristics and the latter locates these characteristics at a specific sequence position. In this way, a certain keyword is only assigned to a sequence if sequence similarity is found in the position described by the FT field. Exhaustive tests performed over sequences with homologues (cluster set) and without homologues (singleton set) in the database show that assigning functions is much 'cleaner' when information about domains (FT field) is used, than when only the keywords are used. Copyright © 2002 John Wiley & Sons, Ltd.**

**Keywords:** **protomotifs; data mining; association discovery; computational function assignment; functional motifs; protein databases**

### Introduction

Automatically discovering knowledge about the content of genomes is one of the most exciting challenges in the post-genomic era. Searching for homologies and evolutionary relationships between sequences is by far the most frequently used strategy for assigning functions to new sequences. An extensive collection of software is available for routine database searches based on heuristic approaches such as FASTA (Pearson and Lipman, 1988), and BLAST (Altschul *et al*., 1990, 1997), or more exhaustive procedures based on dynamic programming algorithms (Needleman and Wunsch, 1970; Smith and Waterman, 1981). High assurance and accuracy are obtained from these methodologies when sequences similar to a given query sequence clearly exist in databases (Agarwal and States, 1998; Brenner *et al*., 1998).

Pattern-based procedures are alternative tools for deriving evolutionary, structural or functional information about a query sequence. Several pattern-matching tools are used to this end over a large collection of specific pattern/domain databases: Prosite (Hofmann *et al*., 1999), Pfam (Bateman *et al*., 2000), Blocks (Henikoff *et al*., 2000), Prints (Attwood *et al*., 2000), ProDom (Corpet *et al*., 2000), SMART (Ponting *et al*., 1999), Domo (Gracy and Argos, 1998), Identify (Nevill-Manning *et al*., 1998), and PROF_PAT (Bachinsky *et al*., 2000). Additionally there are several function annotation algorithms for grouping proteins of similar function by detecting the presence of patterns stored in these databases (Fleischmann *et al*., 1999; Kretschmann *et al*., 2001) and to predict function through the use of well-established patterns (i.e. *diagnostic patterns*).

However, at present, a full solution to the problem of protein functional assignment remains unattainable, especially when working with query sequences which have no clear homologues in

Copyright © 2002 John Wiley & Sons, Ltd.

the sequence databases. In fact, sequences distantly related to the query sequence often fall into the 'twilight zone' (Doolittle, 1986), where by-chance relationships might appear as significant as real ones. Thus, there are still numerous protein sequences whose function remains unknown.

Genes (or proteins) with no matches in the sequence databases are commonly known as *orphans*. Many of them cannot be annotated because their ancestral homologies, or weak relationships, are not detectable by current methodologies.

The modular nature of many proteins can cause additional complications. When matching multidomain proteins it may not be clear which domain(s) correspond to the function associated with each protein. Most of the prediction systems ignore this very important issue, assigning incorrect functions that are linked to the true ones (Karp, 1998). Therefore, developing new automatic strategies addressing the multidomain problem is important for helping in the identification and assignment of specific protein functions.

Most of the current methods used for this aim are based on conventional by-similarity comparison. We propose an alternative system based on detecting small significant fragments by identity that could act as modules in peptide construction. This strategy provides complementary information for addressing the problem of assigning function to a query protein.

So, the method presented assigns functions to peptide regions of protein sequences, approaching the multidomain problem. The method is based on the information contained in protein databases, constituting a particular data mining procedure. The overall procedure is divided into two distinct successive stages. First, a query sequence is analysed to find statistically significant subtle amino acid patterns that are also present in the database (Thode *et al.*, 1996; Rodriguez *et al.*, 2000), here called *protomotifs* (because they do not constitute separate motifs with their own structural or functional organization). This first step is similar to TEIRESIAS (Rigoutsos and Floratos, 1998; Floratos *et al.*, 2001). This algorithm finds patterns of variable length with ambiguous positions, whereas our algorithm locates fixed-length and well-defined ones. We have previously used similarity based searches for detecting small fragments (Rodriguez *et al.*, 2000), but we have found that fixed-length patterns better resemble small but strongly conserved similarities (Thode *et al.*, 1996). In the second step, the protomotifs are associated with the functional annotations derived from the original SWISS-PROT entries that gave rise to them, and then used for assigning functions to the analysed sequence.

Exhaustive tests have been performed to evaluate the usefulness of the whole method. These tests have been performed with different sets of sequences extracted from well-known databases where these sequences are hierarchically organized by similarity levels. The strategy was able to reveal unknown and potentially important information for protein function prediction.

### Material and methods

### Test sets

SWISS-PROT (Bairoch and Apweiler, 2000) is one of the most reliable and best known repositories of protein sequence information (Apweiler, 2001). In addition to the protein sequence, each entry contains valuable data about the function of the protein (keywords; *KW* field) and also concerning the region of the protein in which the function is located (features; *FT* field). We have used SWISS-PROT release 38 (July, 1999), with exactly 80 000 sequences, as the training protein database.

*ProtoMap release 3.0* (Yona *et al.*, 2000) was used to evaluate the method and build the test sets. In this database SWISS-PROT and TREMBL sequences are clustered on the basis of their degree of sequence similarity. An *entrance level* equivalent to E-value = 1 (a low stringency level, which results in a small number of clusters with many sequences each) was used, and only sequences belonging to the SWISS-PROT database were selected, excluding low-complexity sequences, to avoid gathering compositionally biased protomotifs (Bork and Koonin, 1998). Sequences less than 30 amino acids in length were also discarded. In this way we obtained 6692 clusters with more than one sequence, and 1381 clusters with only one sequence (singletons).

Two main sets were defined from this database, as detailed below.

### Cluster set

Five hundred different clusters (250 with some Prosite annotation and 250 with none) were

Copyright © 2002 John Wiley & Sons, Ltd. 

selected as a representative sample of the 6692 clusters. These clusters were selected as the most informative groups, being those with the highest number of functional annotations in the KW and FT fields in their corresponding SWISS-PROT records. In turn, the sequence with the most annotations in each cluster was chosen for analysis as the representative sequence of its group. The two subsets formed from this *cluster* set, according to their Prosite annotation, are respectively called the *cluster-prosite* set and the *cluster-noprosite* set. The first subset was expected to have a greater amount of functional information.

### Singleton set

The 600 sequences with the most functional annotations were chosen as a representative sample of the 1381 non-clustered ProtoMap sequences. This set initially comprised three subgroups of 200 sequences each, on the basis of different E-value ranks (Pearson and Lipman, 1988) (given a score of similarity between a query sequence and one sequence from SWISS-PROT, the E-value is the probability of finding a sequence in the database with that or a better score of similarity):

- *Singleton-1* set: the most significant similarity has $0.01 <$ E-value $\leq 0.1$.
- *Singleton-0* set: the most significant similarity has $0.1 <$ E-value $\leq 1$.
- *Singleton-00* set: the most significant similarity has E-value $> 1$.

It is worth noting that all of them lack similarity, having E-values of more than 0.02 (the upper threshold for significant homologies; Pearson, 1996). Thus, they resemble orphan sequences at different levels of sequence similarity, but since they have functional annotations, it is possible to use them to evaluate the method's predictions.

*SCOP release 1.53* (structural classification of proteins; Murzin *et al.*, 1995) was used to extend and evaluate the possibilities of the method in the area of structure prediction. This database contains all of the known protein structural domains organized in a hierarchical fashion, the main hierarchy levels being (in descending order): *class, common fold, superfamily* and *family*.

In each test experiment, when a sequence is used as a query to predict its function or structure, such a sequence is removed from the database in order to avoid the occurrence of self-homology, which would undermine the criteria used to build the test datasets.

### Obtaining protomotifs

The algorithm created by Thode *et al.* (1996) was used to obtain protomotifs (small significant peptides) associated with functional annotations of their source sequences. A protomotif by itself does not contain enough information to infer a relationship between the sequences sharing it (Rigoutsos *et al.*, 2000). However, the presence of several of these signals in different sequences with common functional descriptors (keywords, features, etc.) strengthens the signal sufficiently to allow a relationship to be expressed. Our novel approach has been used for verifying functional information and for the gene identification problem (Thode *et al.*, 1996).

The algorithm proceeds by searching for fixed-length conserved fragments common to both a query and the database sequences (see Figure 1a for details) with a minimum number of identical residues. Their successive overlapping throughout the query sequence will later extend these short fragments. The best results were obtained when using a 10-residue window length with six identical amino acids as stringency value (Thode *et al.*, 1996).

In this way, all sequence fragments related to each potential protomotif along the query sequence, and its functional information are obtained. A first sieve is carried out to remove by-chance protomotifs, removing all those putative protomotifs that do not satisfy a minimum support (present in at least three sequences) and those that are not statistically significant. For these latter, a *frequency index* (*FI*) is used, which represents the expected probability of a given protomotif being found purely by chance. The *FI* is computed by combining the frequencies of each amino acid belonging to the protomotif:

$$F_{\mathrm{i}} = n \prod_{aa=1}^{6} f_{aa}$$

where $n$ is the number of amino acids in the database and $f_{aa}$ is the absolute frequency of each

Copyright © 2002 John Wiley & Sons, Ltd.

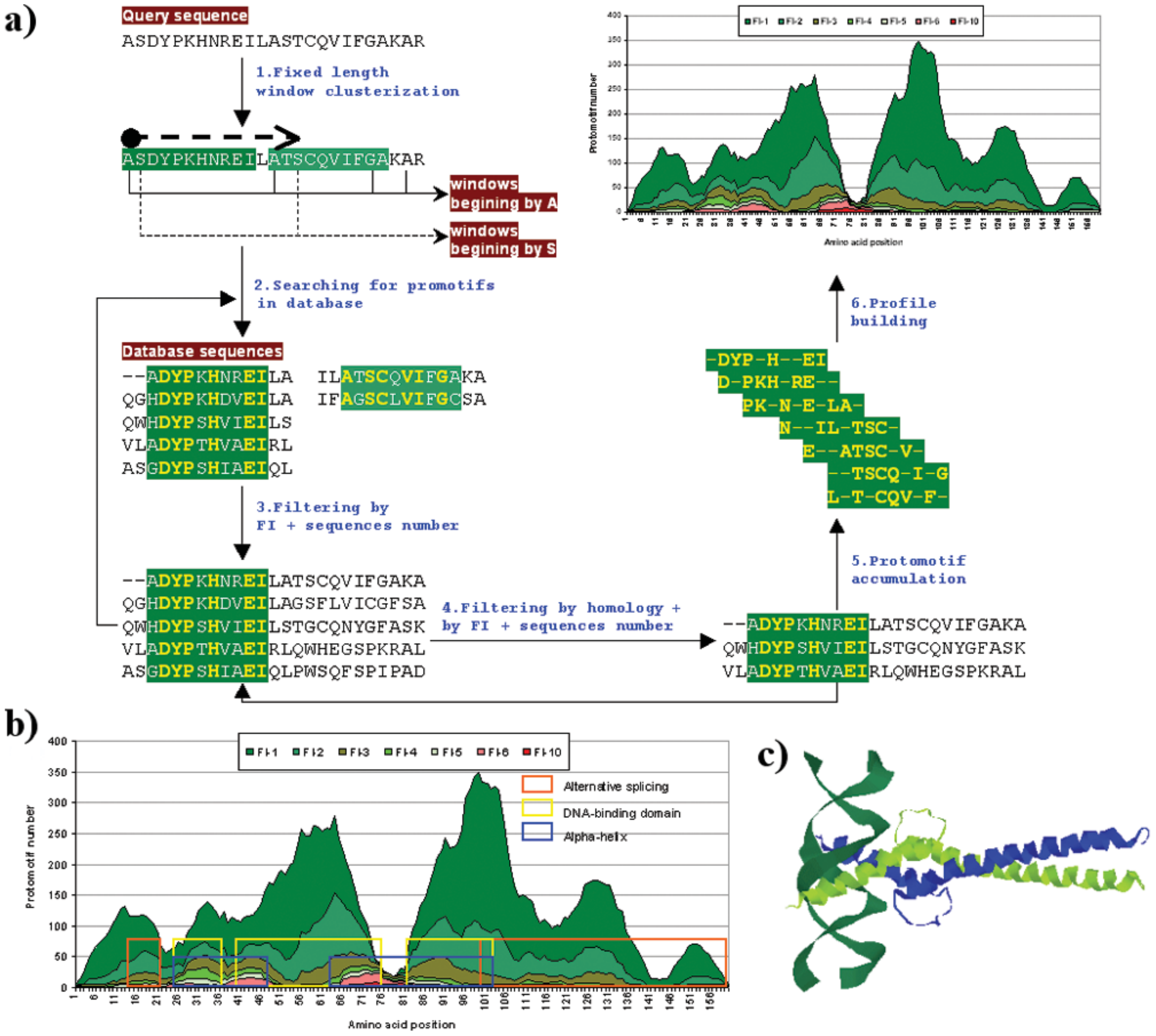

**Figure 1.** Anagram algorithm. (a) Steps carried out to obtain protomotifs from a query sequence: (1) each fragment of fixed length along the query sequence is obtained by using a sliding window and is classified by the first amino acid; (2) each fragment is searched for in the protein database and those with a minimum number of amino acid matches are registered; (3) a minimum number of database sequences is required for a fragment to be taken into account in the process and remaining fragments are evaluated as a function of their statistical significance (FI); (4) any database sequence, containing a fragment, with a minimum level of local sequence similarity with others is removed, avoiding sequence redundancy, and the FI of the remaining fragments is recalculated; (5) the final fragments (now called protomotifs) are accumulated by the query sequence position; (6) finally, a protomotif accumulation profile (PAP) is generated. (b) PAPs corresponding to different significance levels (FIs), for a transcription regulation protein (code SWISS-PROT: MAX_HUMAN). The boxes on the profile represent the different structural/functional domains indicated in the entry from SWISS-PROT. A certain functional correspondence is shown between: the DNA binding domains (of a general type) with the accumulations of lower FI, and more specific regions, such as the two $\alpha$-helices [DNA binding and molecule dimerization in (c)], with the accumulations of higher FI. (c) Structure of MAX protein bound to DNA [PDB code (Bhat *et al.*, 2001) — database of known 3D-structures: 1AN2] highlighting its two subunits (with the two $\alpha$-helices corresponding to the profile in (b) each one) in different colours

Copyright © 2002 John Wiley & Sons, Ltd.

amino acid (aa) in the database. Thus, in conjunction with the observed protomotif frequency in the search ($f_i$), the $FI$ is finally obtained as:

$$FI = \frac{f_i}{F_i}$$

Thus, for $FI > 1$, the pattern will be in the database with a value above that of the expected one, and the higher this index, the lower the probability of finding this pattern by pure chance. A second sieve is carried out to remove similar sequences. Thus, for each pair of sequences sharing a given protomotif a similarity value is computed in a window of 50 amino acids where the protomotif is centred (to avoid the multidomain problem). When the similarity value is higher than a given threshold (45% in our tests) one of the sequences is removed. As this last sieve could modify the initially observed frequency ($f_i$) for a given protomotif, the first sieve is carried out again for the remaining protomotifs.

At this point a collection of statistically significant protomotifs is available for the query sequence. A histogram with the protomotif frequency (i.e. the number of sequences that contain the protomotif in a given position of the query sequence) detected at each query sequence position represents a *protomotif accumulation profile* (PAP) for the sequence (see Figure 1). In this profile, peaks represent conserved zones of the protein and valleys correspond to less conserved zones, therefore the latter could represent transition zones between different domains (Figures 1b and 1c). Thus, the $FI$ value can be used for grouping protomotifs according to a given significance level.

### Function assignment

The immediate use of the protomotif accumulations is the assignment of functions to new proteins. In particular, accumulations of protomotifs matching specific regions in the query protein could provide information to solve this problem. However, complications could arise from the modular nature of many proteins. When matching multidomain proteins it may not be clear which domain(s) correctly correspond to the functional annotations associated with a protein. A way of solving this problem is through the establishment of correlations between general function annotations (keywords) and positional domain annotations (features) on the entries of the protein records, with the aim of assigning keywords that specifically correspond to domains.

To this end, once the PAPs and the sequences related to them (linked to protomotifs) have been obtained, their corresponding functional annotations are taken from the SWISS-PROT database. At first, keyword field (KW) annotations were used due to their great informative content and ease of use, since they constitute a well-defined and controlled vocabulary. However, as was explained in the previous paragraph, the feature field (FT) was also included with the aim of a more selective analysis, since it displays positional information about concrete domains, which allows us to address the multidomain problem. It is important to note that the FT field is organized according to various data items: *key-name* (the group to which the FT belongs), *from* and *to* (the initial and final domain position) which define the limits of a range, and the domain *description*. Examples are presented in Figure 2.

Several FT-KW dictionaries were compiled to describe correlations between keywords and feature fields. In a first step, FT groups (*key name*) related to functions or post-translational modifications (BINDING, CARBOHYD, CA_BIND, CHAIN, DNA_BIND, DOMAIN, LIPID, METAL, MOD_RES, NP_BIND, PEPTIDE, SIGNAL, SITE, TRANSIT, TRANSMEM, ZN_FING) were selected separately and their *description* items were

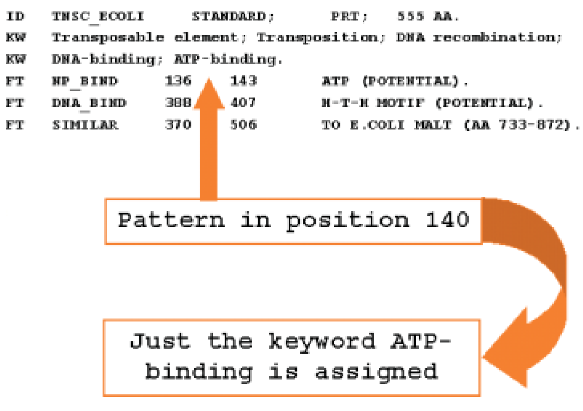

**Figure 2.** Assignment of functional annotations by using the SWISS-PROT *FT* field. In the TNSC_ECOLI record (sequence linked to a protomotif) the match has been found in the 136–143 range, therefore only the keywords related to the *FT* of this range (in this case, ATP-binding) will continue through the later analyses, but not the remaining keywords

Copyright © 2002 John Wiley & Sons, Ltd.

filtered (removing numerical characters, annotations between parenthesis, and some non-informative words) in order to combine equivalent descriptions (e.g. 'EGF-LIKE', 'EGF-LIKE 1', and 'EGF-LIKE INCOMPLETE' were grouped as EGF-LIKE).

Second, to obtain the most significant relations, correlations between keywords and features were computed by considering which keywords appeared to be correlated with a certain feature (support) and in what proportion (confidence) (see Table 1).

Finally, these dictionaries were used in the following way: when a protomotif falls inside the positional range (*from–to*) of a given FT, then only the associated keywords (from the corresponding FT-KW dictionary) are included in the function assignment procedure. Otherwise, if the protomotif does not match any positional annotation of the FT field, only those keywords not associated with any FT field in the protein are included in the later analyses (Figure 2).

Table 1. FT-KW dictionary for the NP_BIND key name. A minimum of five FT-KW relations has been demanded (support), with at least 95% correspondence (confidence). Eleven different correlations (rules) have been identified in this FT group that fulfil support and confidence thresholds, that is, linking an FT to its related KWs. The same feature can be linked to different keywords (boxes)

| Support | Confidence | Rule (*FT* → *KW*) |
|---|---|---|
| 4115 | 1.00 | ATP → ATP-binding |
| 41 | 1.00 | CAMP → cAMP-binding |
| 406 | 1.00 | FAD → FAD |
| 16 | 1.00 | FAD_OR_NAD → FAD |
| 51 | 1.00 | FMN → FMN |
| 51 | 1.00 | FMN → Oxidoreductase |
| 1405 | 1.00 | GTP → GTP-binding |
| 6 | 1.00 | NADH → FAD |
| 6 | 1.00 | NADH → Flavoprotein |
| 6 | 1.00 | NADH → NADP |
| 6 | 1.00 | NADH → Nitrate assimilation |
| 223 | 0.974 | NADP → NADP |
| 225 | 0.982 | NADP → Oxidoreductase |
| 11 | 1.00 | NADPH → NADP |
| 11 | 1.00 | NADPH → Oxidoreductase |
| 81 | 1.00 | NAD OR NADP → Oxidoreductase |
| 8 | 1.00 | PYRIDINE → FAD |
| 8 | 1.00 | PYRIDINE → Flavoprotein |
| 8 | 1.00 | PYRIDINE → Oxidoreductase |

It is worth noting that a given database sequence can contain different FT fields. In this case, it is a sufficient condition for the protomotif to match only one of them for assigning the associated keywords to the analysis. It is also possible that the protomotif does not match any FT field, in which case, only those keywords belonging to the protein but not associated with the FT fields are included in the analysis.

Two types of filters were applied to the SWISS-PROT database to optimize the function assignment procedure. The first filter removes those keywords considered as non-informative, under the criterion that they refer to nucleotide sequence properties of the corresponding protein, or due to their lack of specificity (Table 2). The second filter eliminates sequences containing the 'hypothetical protein' keyword, because they come from a direct translation of potential ORFs, whereas their other keywords are obtained due to their similarity to other protein sequences. This last filter reduces redundancy while maintaining the most informative entries for analysis.

Once the database is filtered, the keywords that belong to all the sequences linked to the protomotifs in the query sequence are collected to form a keyword dataset for the query. For this dataset the relative frequency ($f_k$) of each keyword is computed as:

$$f_k = \frac{n_k}{\sum_{i=1}^{m} n_i}$$

where $n_k$ is the absolute frequency of the keyword $k$ in the dataset and $m$ is the number of keywords in the query sequence. Then the relative frequency of the keyword ($F_k$) in the whole database is

Table 2. Non-specific function keywords in the SWISS-PROT database considered in this work as non-informative

| | |
|---|---|
| 3D structure | Multifunctional enzyme |
| Alternative initiation | Multigene family |
| Alternative splicing | Pharmaceutical |
| Disease mutation | Plasmid |
| Duplication | Polymorphism |
| Early protein | Polyprotein |
| Heptad repeat pattern | Repeat |
| Hypothetical protein | Triplet repeat expansion |
| Late protein | |

Copyright © 2002 John Wiley & Sons, Ltd. 

calculated, and the ratio $f_k/F_k$ can be considered as the *probability ratio* ($P_k$) of the keyword:

$$P_k = \frac{f_k}{F_k}$$

If this ratio is higher than one, then the keyword will be considered significant. It is also important to bear in mind that protomotifs have an associated significance level (FI), which is inherited by the corresponding keywords. In this way the keywords can be organized on the basis of the significance of their corresponding protomotifs and filtered by the significance threshold. All the keywords with significance greater than the specified threshold remain as informative.

Additionally, keywords with a low *nk* value ($nk < 4$ give the best scores with the datasets used; data not shown) are considered as non-significant to avoid such a keyword attracting significance merely by virtue of a very low frequency in the database.

### Evaluation of the method

To estimate the accuracy of the proposed strategy, the *simple matching coefficient* (*SMC*) is calculated from the combination of four parameters (Burset and Guigo, 1996):

$$SMC = \frac{TP + TN}{TP + FN + TN + FP}$$

This coefficient is the probability of correct prediction, which here is the probability of a keyword having the same value as predicted, where:

- *TP* (true positives) = the number of real keyword/sequence relations that have been correctly predicted as significant ones.
- *FP* (false-positives) = the number of false keyword/sequence relations that have been mistakenly predicted as significant ones.
- *FN* (false-negatives) = the number of true keyword/sequence relations that have been mistakenly predicted as non-significant ones.
- *TN* (true negatives) = the number of false keyword/sequence relations that have been correctly predicted as non-significant ones.

*SMC* values close to 0 represent a minimum in the accuracy of the method, since the keyword/sequence relationship would arise by chance, whereas *SMC* values close to 1 represent a near perfect prediction.

However, when the *TN:TP* ratio is very high, results trend to be biased by the high *TN* value (as is the case with biological sequence keywords). In this case, a modification of the correlation coefficient is preferred for measuring the accuracy of the method (Burset and Guigo, 1996). This index is not affected by high *TN* values, and supplies a more precise valuation of results:

$$AC = \left[\frac{1}{4}\left(\frac{TP}{TP + FN} + \frac{TP}{TP + FP} + \frac{TN}{TN + FP} + \frac{TN}{TN + FN}\right) - 0,5\right] x2$$

This index ranges from $-1$ to 1, that is, from less to greater accuracy. The four stated parameters can be used for calculating both the sensitivity ($Sn$) and the specificity ($Sp$) indices for the method (Burset and Guigo, 1996):

$$S_n = \frac{TP}{TP + FN} \quad S_p = \frac{TN}{TN + FP}$$

In this work, the *Sn* index represents the proportion of real keyword/sequence relationships that have been correctly found to be significant by the method, and *Sp* is the proportion of false keyword/sequence relationships correctly predicted as non-significant.

### Implementation

To implement the described methodology, customized computer programs were developed for the first step of obtaining the protomotifs through exhaustive database searching (in C ansi language), and for the second step of defining the functional information associated with each protomotif (in PERL language). The former program creates a file with the protomotifs, and the latter creates a file with the more significant keywords. The running time for analysing an average-size sequence (300 amino acids) is about 20 min on an Alpha AXP, 533 Mhz *Workstation*.

Copyright © 2002 John Wiley & Sons, Ltd. 

### Results and discussion

### Function prediction

To demonstrate the procedure, we present the analysis of the *human calmodulin* protein (SWISS-PROT code: *CALM_HUMAN*) as query sequence. In addition, this work demonstrates the effect of the significance indices, and the ability of the strategy to reproduce prior knowledge.

From this analysis, a set of keywords (with a specific *FI* threshold) is obtained (Table 3). The best results are obtained by a combination of: (a) higher *FI*'s; (b) ordering the results on the basis of $P_k$; and (c) filtering them according to the $n_k$ value. In the example shown, the *myristylation* keyword paradoxically appears in the last test with high $P_k$, although it is not related to the analysed sequence. A deeper examination of the keywords belonging to the eight sequences in which myristylation is present shows that up to five sequences include keywords common to the human calmodulin ones. This means that myristylation is linked to another keyword more directly related to the query sequence. However, this false-positive is avoided in our approach by using information associated with the *FT* field (supplementary information). This is just because these sequences have an '*FT* LIPID' field describing myristylation but the protomotifs do not fall in this field range. This clearly reveals that by using the *FT* information it is possible to exclude non-specific keywords (supplementary information).

For exhaustive analysis, the predictions made by our method were tested with the well-known and annotated functional information in the KW fields on the previously defined test sets. These sets were separately analysed and the keywords with a $P_k$ value greater than one were considered significant. In this way, the *SMC* index as well as the sensitivity (*Sn*) and specificity (*Sp*) values were obtained. As expected, high *FI* thresholds produce a significant improvement in the accuracy of the method (Figure 3a).

When these same analyses were carried out with the singleton-00 set, the results were similar though with a lower level of accuracy (Figure 3b). This is explained by the lower average level of homology for these sequences in the database. However, the number of protomotifs found in the sequences of the singleton-00 set is sufficient to predict their functions. In addition, the performance regarding accuracy with respect to the *FI* is similar to that obtained for the cluster-prosite set.

The specificity of the method is always very high, whereas the sensitivity drops down to 50% when the results of the singleton-00 set are analysed (Table 4). In addition, when the *FT* field is used the sensitivity decreases while the specificity increases, thus resulting in a lower number of false-positives.

As a point of reference, Devos and Valencia (2000) reported sensitivity values lower than 25% matching keywords with an alternative method when the sequence identity is as weak as 20%. The lack of specificity in the keyword function definitions is adduced as the main reason for these poor results. Moreover, when the sequence identity drops below 30–40% the specific function of proteins is not generally conserved (Wilson *et al.*, 2000). However, up to 50% sensitivity is obtained with our procedure, even in the worst case, when working with sequences without homologues in the database according to the standard values of sequence similarity.

So far, the test sets used (singleton-00 and cluster-prosite) represent extreme cases (those with the lowest and highest similarity levels). To observe the accuracy of the method under other conditions, we dealt with the sets with intermediate homology levels.

Results obtained for these sets (cluster-noprosite, singleton-1, and singleton-0) fall between those obtained for the cluster-prosite and singleton-00 sets (Figure 4). Very slight differences in the accuracy rate between the main groups (cluster and singleton sets) are observed, and within the groups the differences are even lower (i.e. between the different subgroups). This result is a good indicator of the robustness of the method, meaning that its accuracy is only slightly affected by the homology level of the query sequences with respect to the database sequences. Remarkably, the method is able to define functions for the cluster-noprosite set, almost as precisely as when working with the cluster-prosite set. It suggests that our method is capable of finding homologies where current methods fail.

These results have been obtained using a keyword probability ratio threshold of 1 ($P_k > 1$). When higher thresholds are used ($P_k \geq 2$), an improvement in the specificity of the method is observed, although the sensitivity is negatively

Copyright © 2002 John Wiley & Sons, Ltd. 

Table 3. Progressive evaluation of keyword significance for the human calmodulin protein (SWISS-PROT: CALM_HUMAN) as an example sequence, only considering the *KW* field annotations. Some keywords are emphasized corresponding to the query sequence (bold) or related to their function (italic), though the latter are not in their *KW* field. The keywords in descending order are shown: (a) for frequency and FI $\geq 1$, (b) for frequency and FI $\geq 7$, (c) for $P_k$ and FI $\geq 7$, (d) as (c) but eliminating keywords that appear five times or less ($n_k \leq 5$)

| (a) Keywords | $n_k$ | $f_k$ | (b) Keywords | $n_k$ | $f_k$ |
|---|---|---|---|---|---|
| Signal | 713 | 3.83 | **Calcium-binding** | 82 | 7.6 |
| ATP-binding | 705 | 3.78 | Transferase | 41 | 3.8 |
| Transferase | 701 | 3.76 | ATP-binding | 33 | 3.06 |
| Transmembrane | 601 | 3.23 | Hydrolase | 30 | 2.78 |
| Hydrolase | 577 | 3.1 | Transmembrane | 29 | 2.69 |
| Glycoprotein | 575 | 3.09 | DNA-binding | 27 | 2.5 |
| Oxidoreductase | 443 | 2.38 | Signal | 26 | 2.41 |
| DNA-binding | 430 | 2.31 | Nuclear protein | 25 | 2.32 |
| Nuclear protein | 364 | 1.95 | *Muscle protein* | 19 | 1.76 |
| *Phosphorylation* | 323 | 1.73 | *Phosphorylation* | 19 | 1.76 |
| Transcription regulation | 277 | 1.49 | Oxidoreductase | 16 | 1.48 |
| **Calcium-binding** | 275 | 1.48 | **Acetylation** | 15 | 1.39 |
| Transport | 223 | 1.2 | Zinc-finger | 15 | 1.39 |
| Lyase | 217 | 1.16 | Metal-binding | 14 | 1.3 |
| Ligase | 188 | 1.01 | Transcription regulation | 14 | 1.3 |
| Transit peptide | 150 | 0.81 | *Myosin* | 10 | 0.93 |
| Protein biosynthesis | 146 | 0.78 | *Serine/threonine-PK* | 10 | 0.93 |
| Mitochondrion | 144 | 0.77 | DNA replication | 10 | 0.93 |
| NAD | 139 | 0.75 | Ligase | 10 | 0.93 |
| Zinc | 133 | 0.71 | Lyase | 10 | 0.93 |

| (c) Keywords | $f_k$ | $F_k$ | $P_k$ | (d) Keywords | $f_k$ | $F_k$ | $P_k$ |
|---|---|---|---|---|---|---|---|
| Cardiomyopathy | 0.09 | 0.003 | 34.596 | **Calcium-binding** | 7.6 | 0.323 | 23.506 |
| SH3-binding | 0.09 | 0.003 | 30.272 | *Myosin* | 0.93 | 0.058 | 15.939 |
| Photoprotein | 0.28 | 0.010 | 26.908 | *Muscle protein* | 1.76 | 0.115 | 15.326 |
| Dental caries | 0.09 | 0.004 | 24.217 | Myristylation | 0.74 | 0.134 | 5.516 |
| **Calcium-binding** | 7.6 | 0.323 | 23.506 | **Methylation** | 0.56 | 0.147 | 3.815 |
| Vitamin D3 | 0.09 | 0.004 | 22.016 | Cell division | 0.74 | 0.216 | 3.427 |
| Protein splicing | 0.19 | 0.012 | 16.492 | Metal-binding | 1.3 | 0.419 | 3.101 |
| *Myosin* | 0.93 | 0.058 | 15.939 | *Serine/threonine-PK* | 0.93 | 0.304 | 3.056 |
| *Muscle protein* | 1.76 | 0.115 | 15.326 | Pyridoxal phosphate | 0.74 | 0.252 | 2.941 |
| Organic radical | 0.09 | 0.006 | 14.246 | **Acetylation** | 1.39 | 0.503 | 2.764 |
| Alkylphosphonate uptake | 0.09 | 0.006 | 14.246 | Zinc-finger | 1.39 | 0.538 | 2.583 |
| Polyamine biosynthesis | 0.09 | 0.007 | 12.109 | DNA repair | 0.56 | 0.230 | 2.430 |
| Luminescence | 0.28 | 0.025 | 11.416 | Protein transport | 0.56 | 0.258 | 2.171 |
| Sulphate transport | 0.09 | 0.009 | 10.091 | DNA replication | 0.93 | 0.433 | 2.146 |
| Hypersensitive response | 0.09 | 0.010 | 9.314 | Ligase | 0.93 | 0.491 | 1.896 |
| Sialic acid | 0.09 | 0.012 | 7.339 | Membrane | 0.83 | 0.465 | 1.785 |
| Embryo | 0.19 | 0.028 | 6.727 | *Kinase* | 0.65 | 0.446 | 1.456 |
| Phosphopantetheine | 0.28 | 0.045 | 6.176 | Transferase | 3.8 | 2.628 | 1.445 |
| Galaptin | 0.09 | 0.015 | 5.907 | NADP | 0.56 | 0.389 | 1.437 |
| Dipeptidase | 0.09 | 0.015 | 5.631 | ATP-binding | 3.06 | 2.144 | 1.426 |

affected. This means that although some true positives (*TP*) are lost, greater confidence can be placed on the remaining keywords. Certainly, a tradeoff between the stringency threshold and false-positives must be taken into account for reliable predictions.

### Keyword accumulation profiles (KAPs)

For a more detailed demonstration of the usefulness of the proposed method on sequences with unknown function and to evaluate its ability to discriminate different domains, all the significant

Copyright © 2002 John Wiley & Sons, Ltd. 

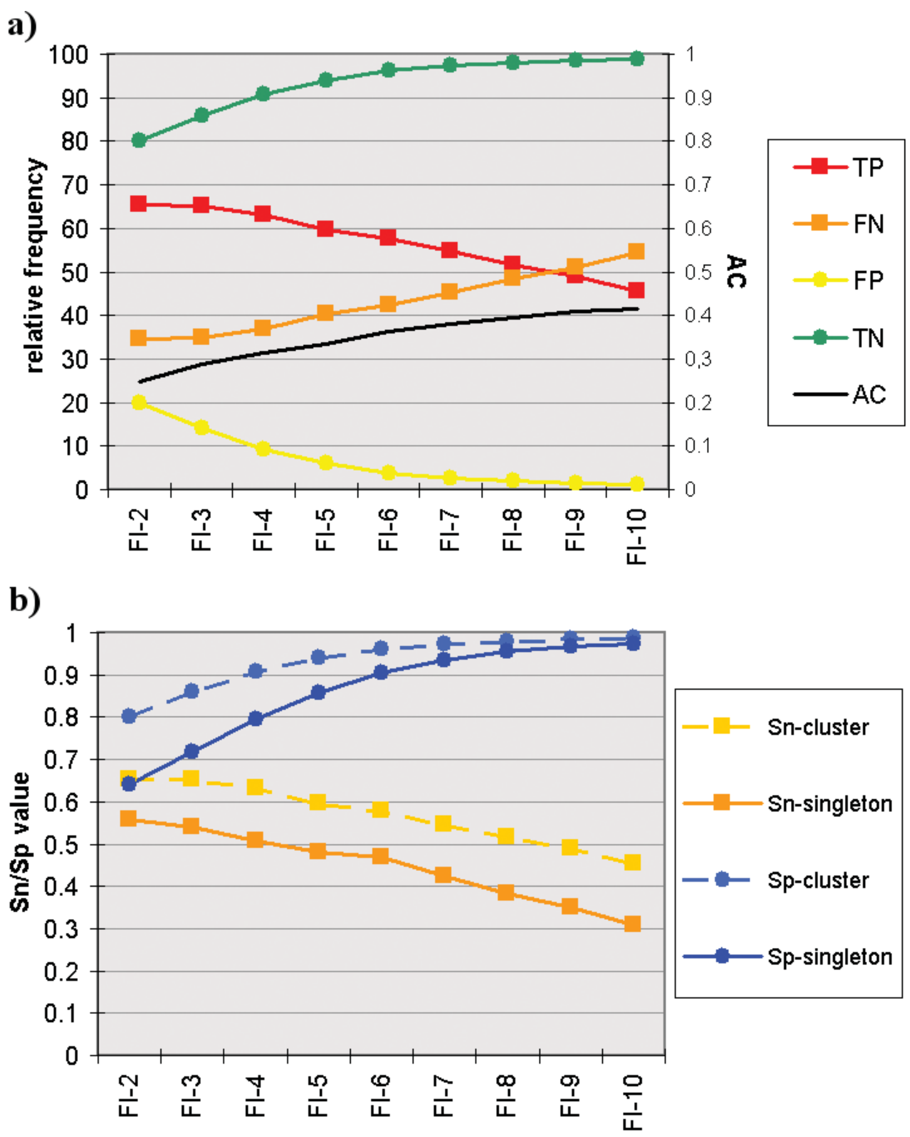

**Figure 3.** The accuracy of the method when the FT field is used. (a) Trends in the number of *TP, FN, FP,* and *TN* for the cluster-prosite set, by increasing the restriction based on different FIs (relative frequencies: *TP*/*FN* and *FP*/*TN*). The AC increases with the FI threshold. (b) Sensitivity (Sn) and specificity (Sp) for both cluster-prosite and singleton-00 sets. Only very slight differences are observed between these sets

Table 4. Values of sensitivity (Sn), specificity (Sp) and SMC for the cluster-prosite and singleton-00 sets. The results to FI-2, FI-10 and all FIs mean value (FI-2 to FI-10) are shown, all of them with and without the use of the *FT* field (*KW* and *KW* + *FT*, respectively)

| | **Cluster-prosite** | | | | | | **Singleton-00** | | | | | |
|---|---|---|---|---|---|---|---|---|---|---|---|---|
| | ***KW*** | | | ***KW* + *FT*** | | | ***KW*** | | | ***KW* + *FT*** | | |
| | **FI-2** | **FI-10** | **Mean** | **FI-2** | **FI-10** | **Mean** | **FI-2** | **FI-10** | **Mean** | **FI-2** | **FI-10** | **Mean** |
| Sn | 80.86 | 81.59 | 84.56 | 65.38 | 45.40 | 56.87 | 61.82 | 38.18 | 51.42 | 53.74 | 21.88 | 38.03 |
| Sp | 60.48 | 85.35 | 74.04 | 79.99 | 98.88 | 93.30 | 54.62 | 80.21 | 67.89 | 57.99 | 92.99 | 78.51 |
| SMC | 0.61 | 0.85 | 0.74 | 0.80 | 0.98 | 0.93 | 0.55 | 0.79 | 0.67 | 0.58 | 0.92 | 0.78 |

Copyright © 2002 John Wiley & Sons, Ltd. 

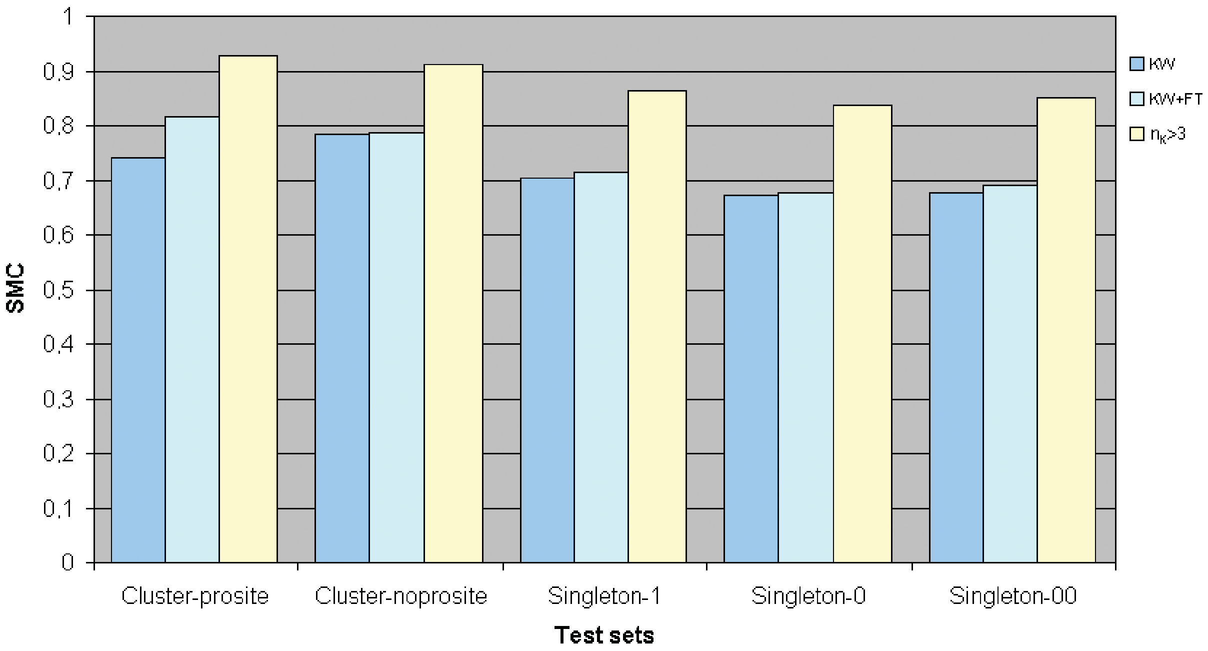

**Figure 4.** SMC value for the different test sets: with *KW*, with *KW* + *FT*, and with *KW* + *FT* and filtering below four appearances by keyword ($n_k > 3$). Note that a higher SMC value corresponds to the last test keywords from several sequences were selected. Then, *keyword accumulation profiles* (KAPs) were constructed by separately accumulating only those protomotifs that contain a specific keyword, to analyse whether accumulations of specific protomotifs are associated with specific keywords.

To illustrate the results, a keyword profile for human coagulation factor IX (SWISS-PROT code: *FA9_HUMAN*) in the cluster-prosite set was compiled. Protomotifs were obtained by comparing this sequence against all the SWISS-PROT sequences. When the *FT* field is not used in the analysis (Figure 5a) the '*EGF-like domain*' keyword profile of this sequence shows nonspecific protomotif accumulations at different regions along the sequence. On the other hand, when the *FT* field is used in the analysis, the results are clearly filtered and the keywords are restricted almost exclusively to those places where the real protein function is defined. It is noteworthy that protomotifs with the '*serine protease*' keyword coming from one subtilisin and several chymotrypsin sequences are found, because all of them are serine proteases, despite presenting different structures and amino acid sequences.

As can be observed, the '*DNA-directed DNA polymerase*' keyword also appears as a result of the initial analysis. However, when representing this false-positive keyword in the profile, the protomotif accumulations appear dispersed throughout the sequence, without a specific concentration (Figure 5b), indicating that the keyword was linked to non-specific protomotifs. Thus, we have an additional criterion for filtering false-positives reported by the initial analysis.

In the hardest case, when working with the singleton sequence set, in spite of their low homology with the database sequences, it is also possible to identify concrete accumulations in the profiles, easily discriminated from the false-positive ones (Figure 5c). Thus, functional information for these sequences can be extracted and a functional pattern for an orphan protein can be defined, without any prior knowledge about them.

A more specific example of a prediction is given here for a protein from the fission yeast, *Schizosaccharomyces pombe* (gene name in *Sz. pombe* GeneDB (**http://www.genedb.org/**): SPCC1906.01). This sequence is evolutionarily conserved; however its exact function remains unknown, though by-similarity searches suggest a mannose-1-phosphate guanyltransferase (Hashimoto *et al.*, 1997). Searching in the PROSITE database supplies additional information: Hexapeptide-repeat

Copyright © 2002 John Wiley & Sons, Ltd. 

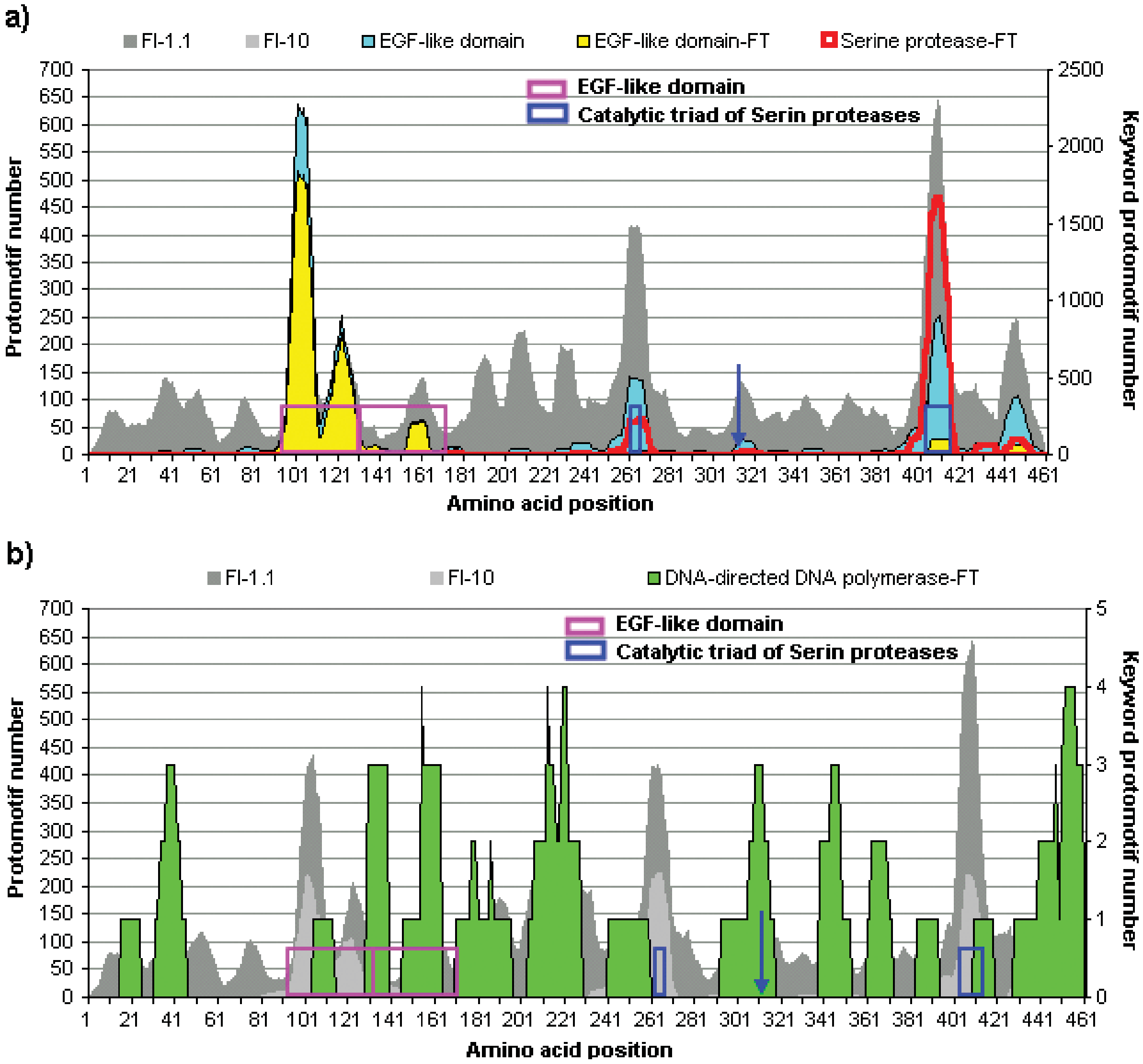

**Figure 5.** Keyword accumulation profiles (KAPs) in the cluster and singleton sets (PAPs in grey in the background). The ordinate axis on the left corresponds to the protomotif profile and the one on the right to the keyword profile. (a) 'EGF-like domain' and 'Serine protease' profiles for the FA9_HUMAN sequence of cluster-prosite set. The boxes represent domains of the protein whose positions are indicated in the SWISS-PROT *FT* field or in Prosite. The arrow corresponds to the third essential amino acid in the catalytic triad of serine proteases (Branden and Tooze, 1991). In addition, the 'EGF-like domain' profile is shown before and after filtering, according to the database domain information. (b) Negative control where the profile for a keyword not contained in the database entry of the FA9_HUMAN protein is shown. Note the smaller scale of the figure (on the right). (c) Keyword profile for the DAP3_HUMAN sequence of the singleton-00 set. All significant keywords for the FI-6 threshold given by the method and having more than 20 appearances are highlighted. The box indicates an ATP-binding pattern, extracted from the FT field in its database entry and also present in Prosite. Note that the ATP-binding profile (the only important accumulation) includes 10 amino acids (typical size of a protomotif) and therefore it does not correspond exactly to the box pattern, which is smaller. (d) KAPs for the SPCC1906.01 protein with all the significant keywords for the FI-4 threshold. Two important peaks appear: one of them within the 45–87 position, whose most significant keywords are Kinase, Cell cycle and Transferase, and another within the 262–278 position again with the Transferase keyword and other keywords related to membrane lipid metabolism

Copyright © 2002 John Wiley & Sons, Ltd. 

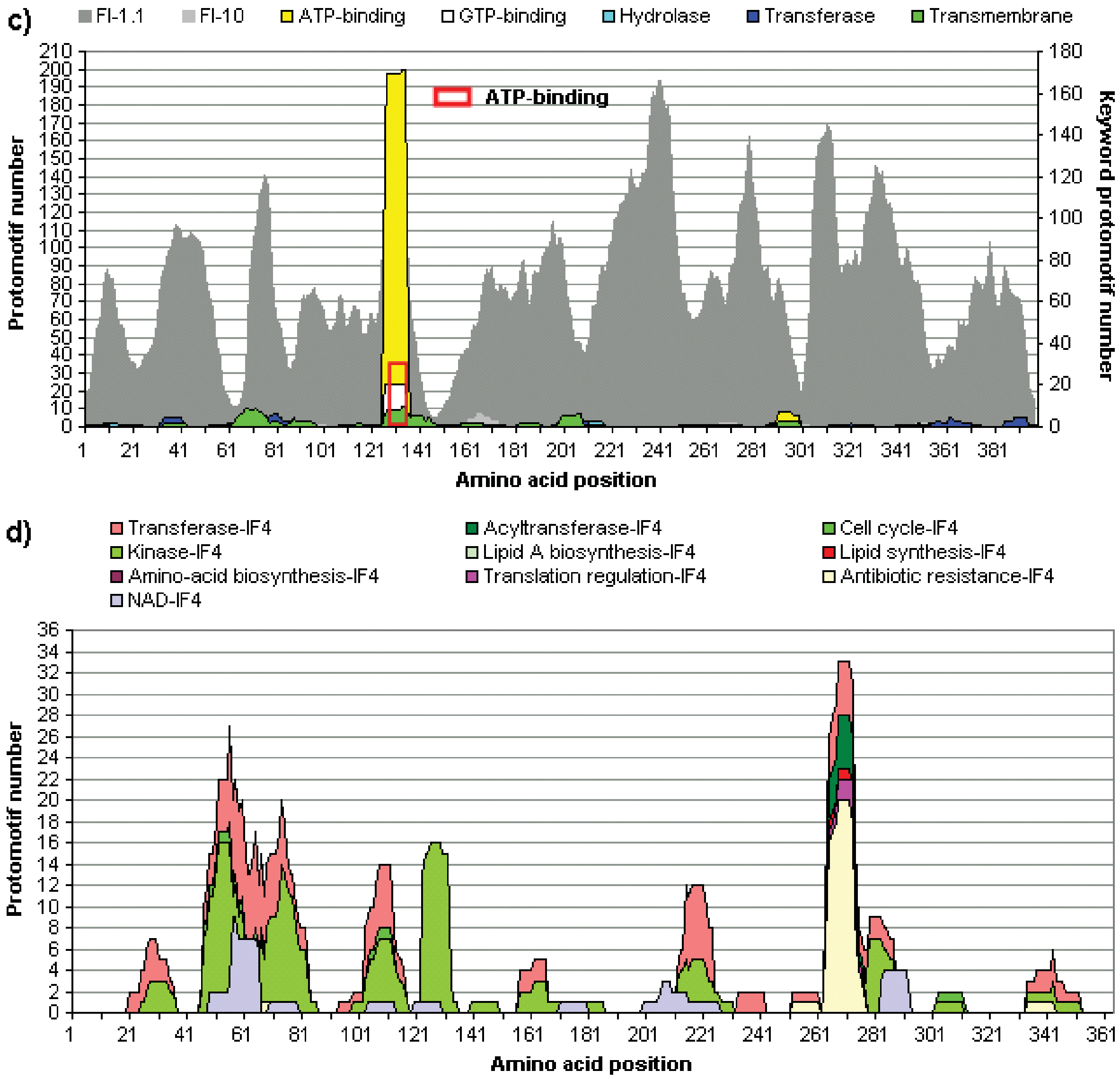

**Figure 5.** Continued containing-transferases signature (258–286 IDPSATIGKNCKIGPNVVIGPNVTIGDGV). This pattern is present in non-specific transferase proteins (including several acetyltransferases, acyltransferases, succinyltransferase and uridyltransferase) with hexapeptide tandem repetitions constituting a structural repeat (Vuorio *et al.*, 1994).

Interestingly, PAKs for this protein (Figure 5d) show the corresponding accumulation in the region described by the Prosite pattern, but with clear dominance of keywords related to membrane lipid biosynthesis. These keywords are not present in the SWISS-PROT entry for this protein. Moreover, all the homologues for this protein, including a paralogue in *Sz. pombe* with 31% identity, share the Prosite pattern and also have similar PAKs (supplementary information). This specific function (acyltransferase) is in agreement with experimental information that describes protein accumulations on the cell poles, near to the plasma membrane (Donoso I, personal communication). This 'cell wall maintenance' role was also mentioned previously for the *Saccharomyces cerevisiae* orthologue (Gellissen and Hollenberg, 1997). Cell poles are

Copyright © 2002 John Wiley & Sons, Ltd.

Table 5. Correspondence values (in percentages) of SCOP hierarchies found in the FI-10 threshold protomotifs for all sequences of known 3D-structures in the test sets with the well-known ones, at the four more important hierarchical levels. (a) cluster-prosite and (b) singleton-00. All sequences that did not have a minimum protomotif support (10) and those that had different structural domains in the same protein sequence have been removed

| (a) | ADAM_ CROAD | BLAT_ ECOLI | C553_ DESVH | CANS_ RAT | FER_ SULS7 | KGUA_ YEAST | PA2_ APIME | PF4L_ HUMAN | SUBB_ BACLE | TETN_ HUMAN | Mean |
|---|---|---|---|---|---|---|---|---|---|---|---|
| Class | 92 | 58 | 90 | 50 | 88 | 25 | 88 | 58 | 98 | 40 | 69 |
| Fold | 85 | 58 | 6.9 | 39 | 88 | 0 | 88 | 25 | 98 | 20 | 51 |
| Superfamily | 85 | 58 | 6.9 | 39 | 88 | 0 | 88 | 25 | 98 | 20 | 51 |
| Family | 1.6 | 58 | 6.9 | 33 | 0 | 0 | 0 | 25 | 98 | 20 | 24 |
| Support | 61 | 12 | 29 | 18 | 50 | 20 | 16 | 12 | 880 | 10 | |

| (b) | COAT_ STMV | EXO_ LAMBD | GFP_ AEQVI | KDNM_ BPT4 | PPOI_ PHYPO | T2BA_ BACAM | Mean |
|---|---|---|---|---|---|---|---|
| Class | 14 | 17 | 0 | 40 | 3 | 55 | 21 |
| Fold | 14 | 0 | 0 | 0 | 0 | 0 | 2 |
| Superfamily | 14 | 0 | 0 | 0 | 0 | 0 | 2 |
| Family | 0 | 0 | 0 | 0 | 0 | 0 | 0 |
| Support | 14 | 12 | 11 | 15 | 29 | 11 | |

Copyright © 2002 John Wiley & Sons, Ltd.

known to be places of cellular growth, and membrane lipid production. This fact supports the predictive capacity of the method and its usefulness in practical situations.

### Structure prediction

In order to analyse correspondences between protomotif accumulations and structural characteristics, two new sets were constructed: one composed of all those sequences from the five previous test sets with references in the PDB database (Bhat *et al.*, 2001) (a database of known 3D structures); and another set composed of all the SWISS-PROT sequences with entries in the PDB database. A protomotif search for all sequences in the first set compared against the second was performed, but in this case the SWISS-PROT functional information was substituted by the hierarchical classification of the SCOP structural domain database (choosing its four first levels: *class, common fold, superfamily* and *family*). Once the results were obtained, these structural hierarchies were compared with those of the known domains of each analysed sequence (Table 5).

As in the function assignment case, the greatest correspondence to the well-known structural information occurs in the more informative of the test sets (cluster-prosite), reaching more than 50% coincidence at the superfamily level. In the singleton-00 set, however, the coincidence percentage at the first level (class) is as low as 21%, decreasing to 2% at the superfamily level. This can be explained by the low number of related proteins that these non-clustered sequences have in the database. In fact, more than 50% of the sequences with a well-known structure in this set do not appear in the table, because they do not have a minimum protomotif support. This conforms to the hypothesis presented in Sander and Schneider (1991) that structural information is strongly conditioned by the identity level of the sequences under analysis. As in the previous case, intermediate values between the extreme sets are obtained for the rest of the test sets (see supplementary information).

Sequences with different structural domains were not directly computed in this analysis, to facilitate the evaluation of the results. However, when SAPs were analysed (similar to those of keywords or KAPs), it was observed that the results were suitably separated according to known different domains of the protein (Figure 6).

It should be stressed that function and structure are not correlated in all cases (Thornton *et al.*, 1999; Hegyi and Gerstein, 1999). However, both protein characteristics always depend on the amino acid sequence. The protomotifs detected by the proposed method could represent evolutionarily conserved function and/or structural relationships.

### Ongoing work

We are currently working on two complementary strategies in order to improve the accuracy of the proposed methodology. First is hierarchical keyword clustering — grouping syntactically different, but functionally related, keywords to corroborate the final results. Work has already been done on the grouping of keywords appearing with a certain frequency in specific protein families (Andrade *et al.*, 1999a). Nevertheless, in this work it is necessary to group keywords which, although they belong to different unrelated sequences, do represent some related structure-function characteristics. Accordingly, keywords such as *ATP-binding, GTP-binding, cAMP-binding* and *cGMP-binding* should belong to a hypothetical '*nucleotide-binding*' class. We have already begun to use a source of keyword clusters (The Gene Ontology Consortium, 2000) and have found relations that did not appear when using the keywords separately (data not shown).

Second, we are working on including additional functional annotations from the protein database, such as biochemical information, indicated by the EC classification (enzyme commission) in the SWISS-PROT DE (description) field. In this work, different keywords have not been discriminated according to their generality or specificity. However, it has been demonstrated that better results are obtained with keywords of specific enzymatic functions than with the most general ones (Devos and Valencia, 2000). Although it should be remembered that there are enzymes with high sequence homology but different precise functions (Gerlt and Babbitt, 2000) that can conserve their substrate specificity, we trust that this protomotif data-mining strategy will be useful for correctly assigning enzymatic functions.

Copyright © 2002 John Wiley & Sons, Ltd.

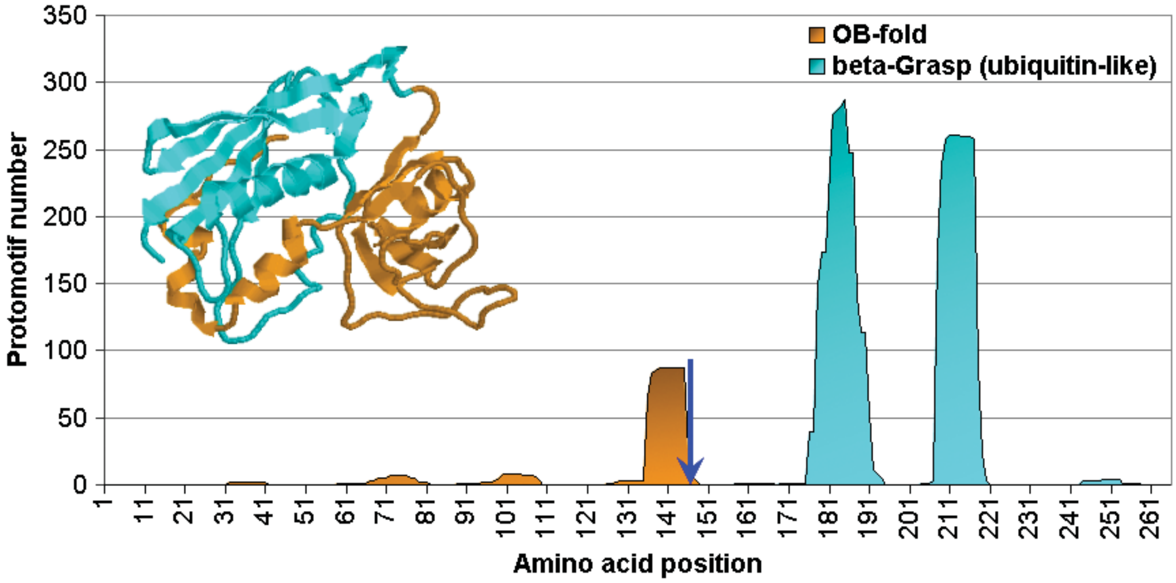

**Figure 6.** Protomotif profiles related to structural domains (SAPs) for the ETC2_STAAU protein using FI-2 protomotifs. Profiles match different domains (2.38 and 4.14) in the 'common-fold' level of the SCOP database. The arrow indicates the separation of both domains. The depicted structure represents the fold for this protein as stored in PDB (PDB code: 1STE) with the same colours corresponding to the profiles of both domains

### Conclusions

In this work a method for assigning functions to proteins is presented, being especially useful when traditional methods do not report any significant homology. The multidomain problem is also approached through a simple and novel methodology supported by functional information available in the *FT* field in the SWISS-PROT database, which enables the location of predicted functions in a specific sequence position.

One of the most important characteristics of the method is the high quantity of information on which it is based, coming from the great number of basic protomotifs that the initial algorithm is able to detect. In addition, this large amount of information is further expanded by the number of keywords that their respective sequences have in the database. This ensures a certain degree of redundancy, which helps to overcome the problem with inconsistency of functional annotation across databases (Brenner, 1999). Other algorithms in the pattern matching application domain, based in well-established peptide patterns (Fleischmann *et al*., 1999; Kretschmann *et al*., 2001) are used to annotate the SWISS-PROT database, with lower sensitivity values than those reported by our methodology. In a similar fashion the approach proposed by Devos and Valencia (2000) is also based on homologous structures already included in hierarchical structural databases, and multidomain proteins are omitted in order to avoid the mutidomain problem. However, our approach is able to reveal new patterns whose presence is useful for predicting accurate functions. In this way, the system supplies enough functional information, even for proteins that do not show similarity to any of the other proteins in the database.

Exhaustive tests have been performed to demonstrate the ability of the strategy to identify previously unknown information. The test sets were designed to cover a broad range of sequences classified by similarity level, including those without significant homologues but with sufficient functional information to verify the predictions. Thus, they could constitute a benchmark for function assignment approaches.

The methodology is able to assign functional keywords with a sensitivity rate higher than 50% on average and a specificity rate near to 90%, so that only one in ten relationships found between a keyword and a protein could be a false one, while one in two real relationships are found on average.

Copyright © 2002 John Wiley & Sons, Ltd. 

In addition, the method is able to discriminate protein sequence regions with defined functions and even structural domains with high accuracy. This data can be used to complement homology searches and function–structure prediction methods, obtaining new knowledge for orphan proteins (Bork and Koonin, 1998).

Although it is not the first time that association discovery methods have been used to order the enormous amount of annotations contained in current scientific databases (even by using the keyword field of the SWISS-PROT database; Devos and Valencia, 2000; Tamames and Tramontano, 2000), most of the prior approaches were based on significant homologies (Guigo and Smith, 1993; Andrade, 1999; Andrade *et al.*, 1999b). However, results reported by these approaches are very scarce when the query protein has no clear homologues in the database. Criteria other than sequence homology searches have also been used (Marcotte *et al.*, 1999; Pellegrini *et al.*, 1999; Eisen, 1998; des Jardins *et al.*, 1997), but these approaches found even less information. It is also noteworthy that our proposed strategy is able to deal with signals coming from other sequence homology-based search engines, to further improve their predictions.

At the present time, as the complete genomes of diverse organisms (including the human genome) are becoming known (The Genome International Sequencing Consortium, 2001; Venter *et al.*, 2001), and observing the revolution that this is giving rise to in medicine and biotechnology, it is necessary to have computational tools available that help us to define the functions of the enormous number of genes that have been discovered. In this context, methods such as the one proposed in this work, in tandem with existing ones, should be of great use in future research.

### Supplementary information

Files with detailed information about the sequence analysis presented in this paper are available at: **http://jaguar.genetica.uma.es**

### Acknowledgements

This work has been partially supported by Grant 1FD97-0372 from the EU-FEDER Programme (Fondos Europeos de Desarrollo Regional) and by Grant QLRT-2000-01473 from the Quality of Life and Management of Living Resources EU Program. We thank Olga Perez for her assistance in statistics, Dr Golan Yona for his inestimable help and essential information for building the test sets, and Juan Antonio Garcia-Ranea and our colleagues in the Genetics Department for their interesting critical review. We are also grateful to Arthur Kerr for his careful reading of the manuscript and valuable modifications.

### References

Agarwal P, States DJ. 1998. Comparative accuracy of methods for protein sequence similarity search. *Bioinformatics* **14**(1): 40–47.

Altschul SF, Gish W, Miller W, Myers EW, Lipman DJ. 1990. Basic local alignment search tool. *J Mol Biol* **215**: 403–410.

Altschul SF, Madden TL, Schaffer AA, *et al.* 1997. Gapped BLAST and PSI-BLAST: a new generation of protein database search programs. *Nucleic Acids Res* **25**(17): 3389–3402.

Andrade MA. 1999. Position-specific annotation of protein function based on multiple homologs. *Proc Int Conf Intell Syst Mol Biol* **99**(7): 28–33.

Andrade MA, Ouzounis C, Sander C, Tamames J, Valencia A. 1999. Functional classes in the three domains of life. *J Mol Evol* **49**: 551–557.

Andrade MA, Brown NP, Leroy C, *et al.* 1999. Automated genome sequence analysis and annotation. *Bioinformatics* **15**: 391–412.

Apweiler R. 2001. Functional information in SWISS-PROT: the basis for large-scale characterisation of protein sequences. *Briefings in Bioinformatics* **2**: 9–18.

Attwood TK, Croning MD, Flower DR, *et al.* 2000. PRINTS-S: the database formerly known as PRINTS. *Nucleic Acids Res* **28**(1): 225–227.

Bachinsky AG, Frolov AS, Naumochkin AN, Nizolenko LP, Yarigin AA. 2000. PROF_PAT 1.3: updated database of patterns used to detect local similarities. *Bioinformatics* **16**(4): 358–366.

Bairoch A, Apweiler R. 2000. The SWISS-PROT protein sequence database and its supplement TrEMBL in 2000. *Nucleic Acids Res* **28**(1): 45–48.

Bateman A, Birney E, Durbin R, Eddy SR, Howe KL, Sonnhammer EL. 2000. The Pfam protein families database. *Nucleic Acids Res* **28**: 263–266.

Bhat TN, Bourne P, Feng Z, *et al.* 2001. The PDB data uniformity project. *Nucleic Acids Res* **29**: 214–218.

Bork P, Koonin EV. 1998. Predicting functions from protein sequences — where are the bottlenecks? *Nature Genet* **18**: 313–318.

Branden C, Tooze J. 1991. An example of enzyme catalysis: serine proteinases. In *Introduction to Protein Structure*. Garland: New York & London; 231–246.

Brenner SE. 1999. Errors in genome annotation. *Trends Genet* **15**(4): 132–133.

Brenner SE, Chothia C, Hubbard TJP. 1998. Assessing sequence comparison methods with reliable structurally identified distant evolutionary relationships. *Proc Natl Acad Sci USA* **95**: 6073–6078.

Burset M, Guigó R. 1996. Evaluation of gene structure prediction programs. *Genomics* **34**: 353–367.

Corpet F, Servant F, Gouzy J, Kahn D. 2000. ProDom and ProDom-CG: tools for protein domain analysis and whole genome comparisons. *Nucleic Acids Res* **28**(1): 267–269.

Copyright © 2002 John Wiley & Sons, Ltd. 

des Jardins M, Karp PD, Krummenacker M, Lee TJ, Ouzounis CA. 1997. Prediction of enzyme classification from protein sequence without the use of sequence similarity. *Proc Int Conf Intell Syst Mol Biol* **5**: 92–99.

Devos D, Valencia A. 2000. Practical limits of function prediction. *Proteins* **41**: 98–107.

Doolittle RF. 1986. *Of URFs and ORFs: a Primer on How to Analyse Derived Amino Acid Sequences*. University Science Books: Mill Valley, CA.

Eisen JA. 1998. Phylogenetics: improving functional predictions for uncharacterized genes by evolutionary analysis. *Genome Res* **8**(3): 163–167.

Fleischmann W, Moeller S, Gateau A, Apweiler R. 1999. A novel method for automatic functional annotation of proteins. *Bioinfomatics* **15**: 228–233.

Floratos A, Rigoutsos I, Parida L, Gao Y. 2001. DELPHI: a pattern-based method for detecting sequence similarity. *IBM J Res and Dev* **45**(3/4): 455–473.

Gellissen G, Hollenberg CP. 1997. Application of yeasts in gene expression studies: a comparison of *Saccharomyces cerevisiae, Hansenula polymorpha* and *Kluyveromyces lactis* — a review. *Gene* **190**: 87–97.

Gerlt JA, Babbitt PC. 2000. Can sequence determine function? *Genome Biol* **1**(5): Reviews 0005.

Gracy J, Argos P. 1998. Automated protein sequence database classification. I. Integration of compositional similarity search, local similarity search, and multiple sequence alignment. *Bioinformatics* **14**(2): 164–173.

Guigó R, Smith T. 1993. Inferring correlation between database queries: analysis of protein sequence patterns. *IEEE Trans Pattern Anal Machine Intell* **15**: 1030–1041.

Hashimoto H, Sakakibara A, Yamasaki M, Yoda K. 1997. *Saccharomyces cerevisiae* VIG9 encodes GDP-mannose pyrophosphorylase, which is essential for protein glycosylation. *J Biol Chem* **272**: 16308–16314.

Hegyi H, Gerstein M. 1999. The relationship between protein structure and function: a comprehensive survey with application to the yeast genome. *J Mol Biol* **288**: 147–164.

Henikoff JG, Greene EA, Pietrokovski S, Henikoff S. 2000. Increased coverage of protein families with the blocks database servers. *Nucleic Acids Res* **28**(1): 228–230.

Hofmann K, Bucher P, Falquet L, Bairoch A. 1999. The PROSITE database; its status in 1999. *Nucleic Acids Res* **27**: 215–219.

Karp PD. 1998. What we do not know about sequence analysis and sequence databases. *Bioinformatics* **14**(9): 753–754.

Kretschmann E, Fleischmann W, Apweiler R. 2001. Automatic rule generation for protein annotation with the C4.5 data mining algorithm applied on SWISS-PROT. *Bioinformatics* **17**: 920–926.

Marcotte EM, Pellegrini M, Thompson MJ, Yeates TO, Eisenberg D. 1999. A combined algorithm for genome-wide prediction of protein function. *Nature* **402**: 83–86.

Murzin AG, Brenner SE, Hubbard T, Chothia C. 1995. SCOP: a structural classification of proteins database for the investigation of sequences and structures. *J Mol Biol* **247**: 536–540.

Needleman SB, Wunsch CD. 1970. A general method applicable to the search for similarities in the amino acid sequence of two proteins. *J Mol Biol* **48**(3): 443–453.

Nevill-Manning CG, Wu TD, Brutlag DL. 1998. Highly specific protein sequence motifs for genome analysis. *Proc Natl Acad Sci USA* **95**(11): 5865–5871.

Pearson WR. 1996. Effective protein sequence comparison. In *Methods in Enzymology: Computer Methods for Macromolecular Sequence Analysis*. Doolittle RF (ed.). Academic Press: New York; 266, 227–258.

Pearson WR, Lipman DJ. 1988. Improved tools for biological sequence comparison. *Proc Natl Acad Sci USA* **85**: 2444–2448.

Pellegrini M, Marcotte EM, Thompson MJ, Eisenberg D, Yeates TO. 1999. Assigning protein functions by comparative genome analysis: protein phylogenetic profiles. *Proc Natl Acad Sci USA* **96**: 4285–4288.

Ponting CP, Schultz J, Milpetz F, Bork P. 1999. SMART: identification and annotation of domains from signalling and extracellular protein sequences. *Nucleic Acids Res* **27**(1): 229–232.

Rigoutsos I, Floratos A. 1998. Combinatorial pattern discovery in biological sequences: the TEIRESIAS algorithm. *Bioinformatics* **14**(1): 55–67.

Rigoutsos I, Floratos A, Parida L, Gao Y, Platt D. 2000. The emergence of pattern discovery techniques in computational biology. *Metabol Eng* **2**: 159–177.

Rodriguez A, Perez-Pulido A, Lopez AD, Thode G, Carazo JM, Trelles O. 2000. Mining Low-level similarity signals from sequence databases. In *Proceedings of the World Multiconference on Systemics, Cybernetics and Informatics (SCI 2000 / ISAS 2000)*, IIIS, vol VIII, Computer Science and Engineering: Part II: Databases. **http://www.iiis.org**

Sander C, Schneider R. 1991. Database of homology derived protein structures and the structural meaning of sequence alignment. *Proteins* **9**(1): 56–68.

Smith TF, Waterman MS. 1981. Identification of common molecular subsequences. *J Mol Biol* **147**: 195–197.

Tamames J, Tramontano A. 2000. DANTE: A workbench for genome analysis. *Trends Biochem Sci* **25**(8): 402–403.

The Gene Ontology Consortium. 2000. Gene Ontology: tool for the unification of biology. *Nature Genet* **25**: 25–29.

The Genome International Sequencing Consortium. 2001. Initial sequencing and analysis of the human genome. *Nature* **409**: 860–921.

Thode G, Ranea JA, Jimenez J. 1996. Search for ancient patterns in protein sequences. *J Mol Evol* **42**: 224–233.

Thornton JM, Orengo CA, Todd AE, Pearl FMG. 1999. Protein folds, functions and evolution. *J Mol Biol* **293**: 333–342.

Venter JC, Adams MD, Myers EW, *et al*. 2001. The sequence of the human genome. *Science* **291**(5507): 1304–1351.

Vuorio R, Harkonen T, Tolvanen M, Vaara M. 1994. The novel hexapeptide motif found in the acyltransferases LpxA and LpxD of lipid A biosynthesis is conserved in various bacteria. *FEBS Lett* **337**(3): 289–292.

Wilson CA, Kreychman J, Gerstein M. 2000. Assessing annotation transfer for genomics: quantifying the relations between protein sequence, structure and function through traditional and probabilistic scores. *J Mol Biol* **297**: 233–249.

Yona G, Linial N, Linial M. 2000. ProtoMap: automatic classification of protein sequences and hierarchy of protein families. *Nucleic Acids Res* **28**(1): 49–55.

Copyright © 2002 John Wiley & Sons, Ltd. 
